# Factors Influencing Postpartum Depression in Nepal: An Integrative Review

**DOI:** 10.31729/jnma.8975

**Published:** 2025-05-31

**Authors:** Jaya Rijal, Satya Rijal, Radhika Upadhyay

**Affiliations:** 1 Nelda C. Stark College of Nursing, Texas Woman's University, Texas, USA; 2 Michigan State University, Michigan, USA; 3 Maharajgunj Nursing Campus, Maharajgunj, Kathmandu, Nepal

**Keywords:** *associated factors*, *Nepal*, *postpartum depression*, *risks*

## Abstract

Postpartum depression remains a critical concern in Nepal, influenced by various socio-cultural and healthcare-related factors. Therefore, this integrative review aimed to analyze and synthesize its key determinants within the Nepalese context. Fourteen articles met the eligibility criteria after systematically searching five electronic databases. The review underscores that increased age, low socioeconomic status, limited educational attainment, unintended pregnancies, inadequate support, and maternal health issues are the prominent risks. Cultural inclinations, such as a preference for male children and lack of adequate antenatal care, contribute to the severity of the condition. The findings stress the need for culturally sensitive strategies, early screening efforts, and the assimilation of mental health care and maternal services. Iwt advocates for focused interventions and future longitudinal studies, and the insights are valuable for healthcare professionals, policymakers, and future investigations to improve maternal health outcomes in Nepal.

## INTRODUCTION

Postpartum depression (PPD) is a subtype of major depressive disorder that occurs within four or six weeks postpartum.^1,2^ Symptoms include sadness, loss of interest, appetite and sleep changes, anxiety, guilt, and, in severe cases, suicidal thoughts or psychosis that may last till a year of childbirth.^3^ Depression during the postnatal period is a growing concern in Nepal, with prevalence reaching up to 39%.^4^ It negatively affects maternal well-being, increasing risks of physical health issues, social isolation, and strained relationships. Additionally, mother-infant bonding gets disrupted, impacting the child's emotional, cognitive, and behavioral development.^5^

Despite numerous studies on PPD determinants in Nepal, findings remain inconsistent, with variations in its risk and protective factors. Given these discrepancies, this integrative review consolidates existing evidence to provide more precise insights into factors influencing PPD in Nepal.

## METHODS

The integrative review method synthesizes diverse research methodologies, including experimental and non-experimental approaches, to provide a comprehensive perspective on a topic. It goes beyond summarizing studies by identifying patterns, discrepancies, and gaps in literature, revealing common themes and emerging concepts. This approach offers valuable insights for researchers, practitioners, and policymakers.^6^

This integrative review included the original full-text, peer-reviewed research studies in English published from 2019 to the date regarding factors associated with PPD in Nepal. The other inclusion criteria encompassed observational studies featuring data collection extending from childbirth to one year postpartum, exclusively among female participants. Studies that specifically targeted certain races, ethnicities, and demographic groups and had mothers with preexisting medical conditions and complications were excluded.

Five electronic databases were searched to identify the studies relevant to the set criteria: PubMed, CINAHL, SCOPUS, Academic Search, and Medline, from July 2024 to January 2025. The MeSH terms used were ("risks" OR "risk factors" OR "contributing factors" OR "pre-disposing factors" OR "determinants" OR "influencing factors") AND ("postpartum depression" OR "depression, postpartum" OR "postnatal depression" OR "PPD" OR "PND" OR "postpartum depression" OR "postnatal depression") AND ("Nepal" OR "Nepalese" OR "Nepalese context"). Finally, the references of extracted studies were screened for their eligibility to be included.

**Figure 1:**
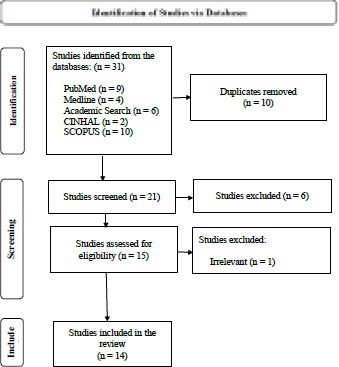
PRISMA flow diagram showing the study selectio n proce ss.

This review was confined to cross-sectional studies. Therefore, the PRISMA flow diagram, derived from the Preferred Reporting Items for the Systematic Review and Meta-Analysis (PRISMA) guideline,7 was employed to ensure a comprehensive review framework. The process had three distinct phases. First, 31 studies were obtained from the initial search of five electronic databases, which were screened for duplicates. Ten duplicate studies were eliminated. The next step was sorting the studies based on the titles and abstracts, which excluded six studies. The remaining 15 were assessed against the eligibility criteria. Finally, 14 studies were retrieved. The PRISMA flow diagram illustrates the entire process (Figure 1).

The details of the data from the finalized studies were extracted and plotted in an Excel spreadsheet. The information included authors, year of publication, region of the country, the aim of the study, sample size, point of data collection, measurement tools, research outcomes, and limitations. There were discrepancies in the design of the studies enrolled, the measurement tools, and the cut-off scores for depression. Thus, the studies were heterogeneous, and the data were synthesized complying with a narrative, thematic approach.

## RESULTS AND DISCUSSION

All 14 extracted studies were descriptive, with one studyhaving a mixed-method design. Six studies were from the central part of Nepal. Most of the studies were published in 2020. The Edinburgh Postnatal Depression Scale (EPDS) was the measurement tool utilized in almost all the studies (Table 1). However, cut-off scores for depression varied. Data was collected from day one to one year after childbirth. Table 1 shows the characteristics of the studies included in the integrative review.

Five primary themes were obtained to summarize the factors influencing PPD: 1) socio-demographics, 2) parental preferences and pregnancy intent, 3) support network, 4) maternal health problems, and 5) access to antenatal care.

**Table 1:** Study characteristics (n = 13).

Authors, Year	Region of the country	Design	Measurement tool	Sample size	Point of data collection	Significant factors of PPD
Priyanka et al.^4^	Western	Cross-sectional	EPDS (cut-off: 12 or more)	218	Within 12 weeks of childbirth	Risks: nuclear family, husband consuming alcohol, and preference of boy child. Protective factors: planned pregnancy, vaginal delivery, male baby, and presence of husband throughout the pregnancy.
Chalise et al.^8^	Central (Bharatpur)	Cross-sectional	EPDS (cut-off: 12 or more)	242	Within 6 months of childbirth	Risks: mother's age over 25 years, smoking, pressure to conceive, unintended pregnancy, and delivery related complications.
Pradhananga et al.^9^	Central (Kathmandu)	Cross-sectional	EPDS (cut-off: 12 or more)	348	6 to 10 weeks of childbirth	Risks: older women (3645 years), and employed mothers.
Tripathi et al.^10^	Eastern	Cross-sectional	EPDS (cut-off: 13 or more)	178	6 to 14 weeks postpartum	Risks: age >30 years, unpaid work, multiparity, and marital dissatisfaction. Protective factors: education above secondary level, monthly income > 40,000 NPR per month, no problems during pregnancy and delivery, no stressful life events in the previous year.
Wasti et al.^11^	Central (Kathmandu)	Cross-sectional	EPDS (cut-off: 10 or more)	300	Third trimester to 7 days postpartum	Risks: unsupportive family members, postnatal period, complication during delivery, history of intimate partner violence, and 1st pregnancy at age 25 or less.
Bhusal et al.^13^	Western (Rupandehi)	Cross-sectional	EPDS (cut-off: 13 or more)	173	< 6 weeks postpartum	Risks: mother with female child, unplanned pregnancy, and pregnancy induced health problems. Protective factors: mother with formal education, whose spouse has secondary level education or more, 4 or more ANC visits.
Chalise & Bhandari^14^	Central (Lalitpur)	Cross-sectional	EPDS (cut-off: 13 or more)	195	Within 6 months of childbirth	Risks: Low educational level, employed in agricultural service and daily wage labor, unwanted pregnancy, lack of support from husband and family, and pregnancy related problems.
Dawadi et al.^15^	Central (Bharatpur)	Cross-sectional	EPDS (no cutoff given)	160	1month to 1 year postpartum	Risks: mother's education level 10 or less than 10th grade, and chronic disease in a family
Ojha & Bhandari^16^	Western (Pokhara)	Cross-sectional	EPDS (cut-off: 13 or more)	172	Within 7 days of childbirth	Risks: household wealth indexes (financial stress, poverty), and pregnancy related problems/complications.
Singh et al.^17^	Southern	Cross-sectional	EPDS (cut-off: 10 or more)	415	Within 10 weeks postpartum	Risks: <150 USD monthly family income, husband migrated for employment, nearest health facility in > 60 minutes of walking distance, C-section, < 4 antenatal checkups. Protective factors: planned pregnancy.
Khadka et al^18^	Central (Ramechhap)	Cross-sectional	Patient Health Questionnaire-2	380	2 months to a year of childbirth	Risks: no postnatal care, nuclear family, living in rural area, having a male baby, complications after delivery, and introducing complementary food to the baby before 6 months of age.
Adhikari et al.^19^	Western (Surkhet)	Cross-sectional	EPDS (cut-off: 13 or more)	347	6 - 12 weeks postpartum	Risks: mother with female baby, history of abortion, and unplanned pregnancy.
Karki & Gurung^23^	Western (Pokhara)	Cross-sectional	EPDS (cut-off: 10 or more)	217	Within a year of childbirth	Risks: Lack of family support and partner support
Neupane et al.^24^	Central (Kathmandu)	Cross-sectional with mixed-method approach	EPDS (cut-off: 12 or more	271	Within 10 weeks postpartum	Risks: painful pregnancy and postpartum experiences, lack of family and spousal support, and superstitions. Protective factors: coping mechanism (self-counseling, self-realization).

## SOCIO-DEMOGRAPHICS

The socio-demographics include the mother's age, education, employment, and economic status. Chalise et al. found higher PPD odds in mothers over 25,^8^ while Pradhananga et al. identified susceptibility in those aged 36-45,9 and Tripathi et al. in those over 30.^10^ In contrast, Wasti et al. reported a greater likelihood of depression in first-time mothers under 25,^11^ which is consistent with a systematic review.^12^ The systematic review's authors presumed that younger mothers are financially weak and cannot cope with the crisis efficiently. However, delayed childbearing challenges and societal judgment in the Nepalese cultural context may have exacerbated emotional distress. Likewise, mothers with formal education and spouses with at least secondary education had lower chances of experiencing depression.^13^ Chalise & Bhandari determined low education as an influencing factor.^14^ Dawadi et al. found vulnerability in those with education up to grade 10,15, which aligns with Tripathi et al.,^10^, who noted higher education as protective. Similarly, mothers in agriculture, daily wages,^14^, and unpaid jobs were more prone to depression.^10^ However, Pradhananga et al. found employment increased susceptibility.^9^ Additionally, income was another predictor. Financial stress, poverty,^16^ and income below $150/month increased the risk,17 while a family income over 40,000 NPR minimized it.^10^ A similar trend was observed in the Middle East, where low family income, limited education, and being a housewife were related to PPD.^12^ Authors claimed that disturbed socioeconomic status, including education, can hinder financial independence, empowerment, and effective coping strategies.

## PARENTAL PREFERENCES AND PREGNANCY NTENT

While a single study determined that having a male baby was linked to PPD,^18^ three studies revealed that those who had a female baby and preferred a boy child were often victims.^4,13,19^ The finding aligned with the study conducted in India, which was likely influenced by Nepal's male-dominated patriarchal society.^20^ Furthermore, unintended pregnancy and pressure to conceive were identified as antecedents of puerperal depression.^4,13,14,17,19^ The current result was consistent with the findings in Ethiopia and the Middle East,^12,21,22^ which was probably because of the constraints in physical and psychological preparedness for childbirth and coping in developing countries.

## SUPPORT NETWORK

Eight studies in this integrative review showed a significant association between the support system available to mothers and PPD. Four studies reported that lack of support from spouses and family members during the postpartum period increased the probability of depression.^14^-^23^-^24^-^25^ Two studies found higher rates among mothers in nuclear families and those with alcoholic husbands while having a supportive spouse throughout pregnancy was beneficial.4,18 Depression was also more prevalent among mothers whose husbands migrated for work,^17^ those experiencing marital dissatisfaction,^10^ and those with a history of intimate partner violence.^25^ These findings align with previous comprehensive reviews.^12,26,21,27^ Motherhood comes with altered roles and responsibilities, disrupted sleep and appetite, and ongoing bodily changes,^28^ making a strong support network essential. Postpartum traditions in Asian communities enhance social support, leading to positive outcomes.^29^ However, the fact that Asian women often refrain from seeking emotional support, which probably increases the chances of PPD, should be considered.

## MATERNAL HEALTH PROBLEMS

Similar to a systematic review,^12^ seven studies in the current review highlighted the impact of pregnancy, delivery, and postpartum health issues on PPD. Three focused solely on pregnancy-related complications,^13,14,16^ two on delivery,^8,18^ and three on complications during both pregnancy and postnatal or pregnancy and delivery.^10,24,25^ Likewise, a significant association was found between the mode of childbirth and PPD. Zhao & Zhang's findings align with the studies of this review.^27^ Mothers who had C-sections were challenged by puerperal depression,^17^ while those with vaginal deliveries had lower chances.^4^ This is likely due to the mother's traumatic experience that imposes additional stress.

## ACCESS TO ANTENATAL CARE

Studies in this review emphasized the importance of antenatal care in reducing PPD odds. Bhusal et al. found that mothers with four or more antenatal visits were less likely to develop PPD. Fewer than four visits, a healthcare facility over 60 minutes away, and those residing in rural areas were ruled as contributing factors.^17,18^ As recommended by these studies, the quality of antenatal care, including frequent checkups and involvement in prenatal educational sessions, can be a medium for expressing emotions and resolving one's queries and concerns. However, accessibility to antenatal health services is limited in Nepal.^30^

## LIMITATIONS

Integrative reviews involve extensive use of literature and are widely used in research. While strategies to enhance data collection and extraction have been developed for the integrative review, there is still a need for further refinement in the analysis, synthesis, and concluding methods, which might have compromised the results. Likewise, the studies included in this integrative review were confined to peer-reviewed articles in English, and none of the grey literature was utilized. Thus, evaluation and exploration of more influencing factors were restricted. Although all the finalized studies used EPDS, the demarcation of scores to diagnose PPD differs, which might have biased the synthesis of the results. Finally, the studies included were not from all regions of Nepal, which makes generalizability questionable.

## IMPLICATION FOR PRACTICE AND FUTURE RESEARCH

Understanding the factors contributing to PPD in Nepal enables healthcare professionals to provide culturally competent care. Integrating cultural beliefs, values, and practices into assessments, interventions, and education ensures sensitivity to women's needs. This knowledge aids in accurate screenings, early identification, and timely interventions. Tailored support can be implemented, such as culturally appropriate counseling and community-based resources. Given Nepalese culture's emphasis on family and community support, involving families in education and treatment fosters a stronger support system.

Recognizing these influences empowers providers to advocate for policy improvements, raise awareness, and promote equitable mental health services. Future research should focus on developing culturally specific interventions and examining long-term outcomes. Longitudinal studies can highlight lasting effects and intervention needs while exploring healthcare disparities can address barriers to quality care.

## CONCLUSION

The evaluation of 14 cross-sectional studies in this integrative review derived five themes, and multiple factors influencing PPD were identified. Low socioeconomic status, limited education, unintended pregnancy, lack of perceived support, maternal health complications across prenatal, intra-natal, and postnatal stages, and inadequate antenatal services are modifiable risks and are preventable. Therefore, healthcare professionals are suggested to apply measures to mitigate and overcome these risks. Similarly, frequent early screening of the mothers is recommended to address the non-modifiable dangers, such as increased age of the mother. Although the preference for male babies requires long-term societal transformation, the vulnerable group of women needs special attention and consideration. To reduce PPD rates and promote puerperal health in Nepal, the integration of maternal and mental health services is deemed necessary.
